# When the Ileum Mimics Appendicitis: A Case of Uncomplicated Ileal Diverticulitis

**DOI:** 10.7759/cureus.98034

**Published:** 2025-11-28

**Authors:** Hanane Aksim, Bah Alpha Amadou, Oualid Haddadia, Meryem Belhamdiya, Rachid Akka

**Affiliations:** 1 Hepatogastroenterology, Military Hospital of Avicenne, Marrakech, MAR

**Keywords:** conservative treatment, differential diagnosis, ileal diverticulitis, surgical treatment, uncomplicated

## Abstract

Although colonic and duodenal diverticulosis is common, ileal diverticular disease is rare. The pathophysiology of ileal diverticula is poorly understood, but the most widely accepted hypothesis currently suggests abnormalities in the myenteric plexus or smooth muscle, high intraluminal pressure, and intestinal dyskinesia. Diagnosis is often delayed and difficult because the clinical presentation is nonspecific, and it is mainly imaging that allows the diagnosis to be established and guided. In the event of complications such as perforation, it can be life-threatening because of delays in diagnosis and treatment.

We present an interesting case of ileal diverticulitis that posed a diagnostic and treatment challenge. A 76-year-old man presented to the emergency department with severe pain in the right iliac fossa that had been developing for 5 days. Once diagnosed, ileal diverticulitis must be managed according to the same therapeutic principles as sigmoid diverticulitis, as was the case for our patient. A misdiagnosis, with low prevalence and nonspecific symptoms, can lead to errors or delays in treatment. Our case shows the importance of having an accurate diagnosis of acute gastrointestinal pathologies before beginning treatment.

## Introduction

Acute terminal ileal diverticulitis is a very rare condition. The prevalence of ileal diverticula has been reported to range from 0.06% to 1.3% [1]. Patients with ileal diverticula are often asymptomatic. When ileal diverticulosis develops into diverticulitis, it usually manifests as tenderness and acute pain in the right iliac fossa. Ileal diverticulitis is often misdiagnosed as acute appendicitis [2]. Non-Meckel's ileal diverticulosis consists of acquired false diverticula, composed of serosa, submucosa, and mucosa. In contrast, Meckel's diverticula are true congenital diverticula that contain all layers of the intestinal wall, including the muscular layer. Recent advances in imaging, particularly CT, have made it possible to diagnose small bowel diverticulitis and differentiate it from appendicitis [3-5]. Acute ileal diverticulitis can be successfully managed with non-surgical treatment [6-8]. Accurate and timely diagnosis of ileal diverticulitis is essential for rapid and close management, as early surgical treatment may be necessary in the event of complications such as obstruction or perforation.

We report the case of a 76-year-old patient who presented with sepsis secondary to acute ileal diverticulitis, which was successfully treated with medical management. This rare clinical case highlights that conservative medical management can lead to successful outcomes in acute ileal diverticulitis.

## Case presentation

We report the case of a 76-year-old man who was admitted to the emergency department with severe cramp-like abdominal pain in the right iliac fossa, without radiation, which had been developing for 5 days and was associated with postprandial vomiting and intermittent episodes of diarrhea without other associated digestive or extra-digestive manifestations. The patient had a history of type II diabetes mellitus under treatment. Clinical examination revealed a conscious patient (Glasgow Coma Score (GCS) 15/15), tachycardia (heart rate (HR) 110 beats/min), normotensive, slightly tachypneic (respiratory rate (RR) 22 cycles/min), and febrile (38.4 °C). Defense and abdominal tenderness were found in the right iliac fossa, with no other specific pathological signs. Laboratory tests revealed leukocytosis at 14,580/mm^3^, C-reactive protein (CRP) at 122.7 mg/L, no electrolyte disturbances, and liver and kidney function tests showed no abnormalities (Table 1).

**Table 1:** Laboratory findings in our case

Parameters	Patient value	Reference range	Interpretation
White blood cell count (/uL)	14580	4000-11000	Markedly elevated
Eosinophilic count (/uL)	60	20-630	Normal
Lymphocytic count (/uL)	1500	1000-4800	Normal
Neutrophilic count (/uL)	11890	1400-7700	markedly elevated
Hemoglobin (g/dl)	11,4	13-18	mildly decreases
Alanine aminotransferase (IU/l)	56	<65	Normal
Aspartate aminotransferase (IU/l)	44	<50	Normal
Alkaline phosphatase (IU/l)	89	40-129	Normal
Gamma-glutamyl transferase (IU/l)	61	8-61	Normal
Total bilirubinemia (umol/l)	11	<17	Normal
Creatinine (umol/l)	80,7	60-120	Normal
Natremia (mmol/l)	134	136-145	Normal
Kaliemia (mmol/l)	4,00	3,5-4,6	Normal
C-reactive protein (mg/l)	122,7	<5	Markedly elevated
Albumin (g/l)	25	35-50	mildly decreases

**Figure 1:**
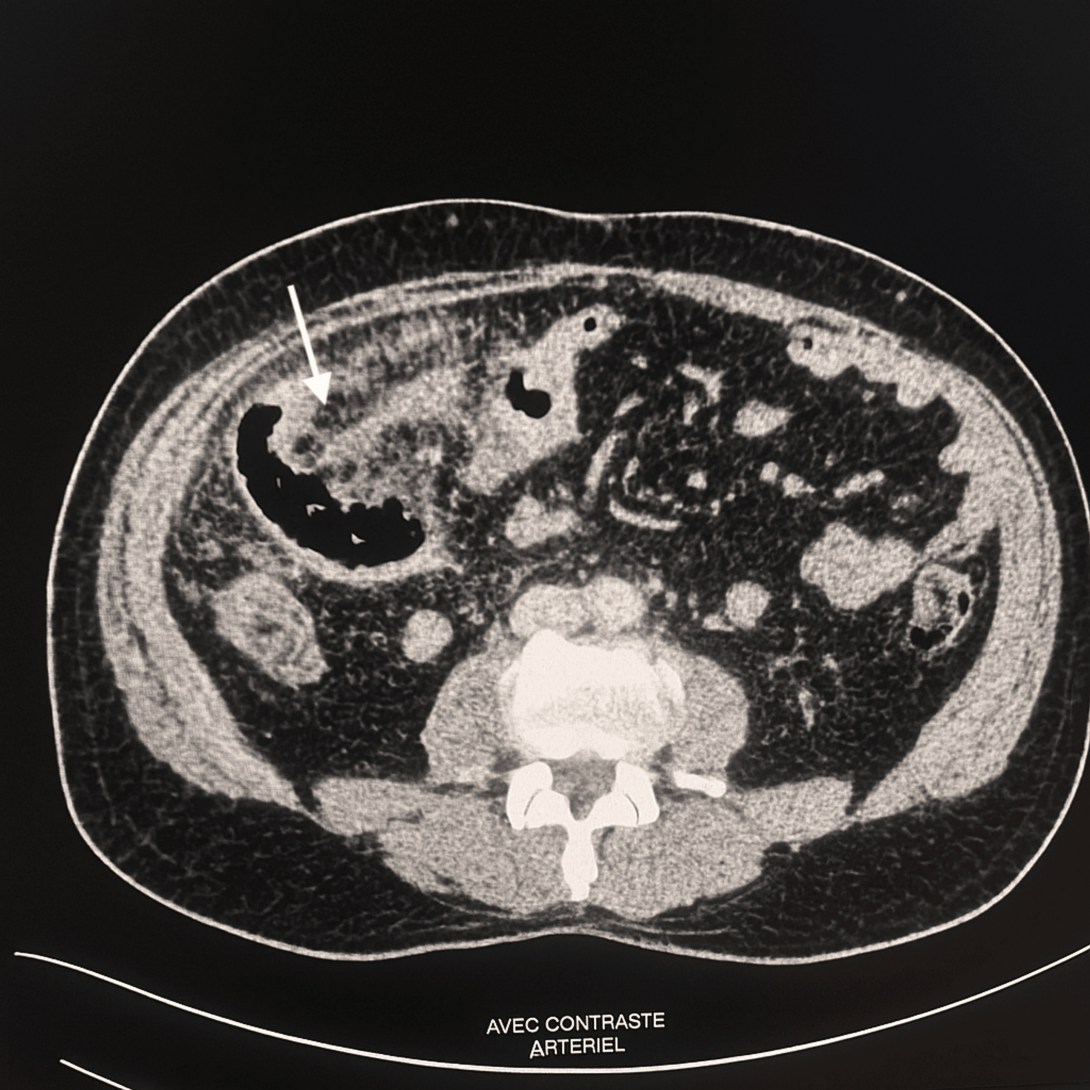
An axial slice from an abdominal CT scan with arterial contrast injection showing terminal ileal diverticulitis (arrow)

The emergency physician suspected acute appendicitis. We completed the assessment with an abdominal ultrasound, but no specific results were found. An abdominal CT scan with contrast was then requested, revealing parietal thickening of the last ileal loop and infiltration of the surrounding fat in several ileal loops, suggestive of ileal diverticulitis (Figure 1), with the presence of uncomplicated colonic diverticulosis. The diagnosis of ileal diverticulitis was confirmed.

The patient was treated with dual intravenous antibiotic therapy consisting of 2 g/day of third-generation cephalosporin and 500 mg x 3/day of metronidazole, combined with symptomatic treatment. The patient's condition improved, with a decrease in infectious markers and a drop in temperature. After five days of intravenous treatment, the patient was allowed to complete the treatment orally and was discharged with follow-up consultations.

## Discussion

Ileal diverticulosis, more specifically, terminal ileal diverticula, is very rare. Ileal diverticula affect men twice as often as women, and the majority of patients are between 60 and 70 years of age [9]. Diverticula tend to be fewer and smaller as one moves from the proximal to the distal part of the small intestine. Diverticular disease occurs predominantly in the proximal jejunum (75%), less frequently in the distal jejunum (20%), and is rare in the ileum (5%) [10]. Coexisting diverticula can occur in the esophagus (2%), stomach (2%), duodenum (15-42%), colon (35-75%), and in the bladder in about 12% of cases [11]. Ileal diverticulosis can be classified into two types: congenital diverticula (such as Meckel's diverticulum) and acquired diverticula.

Unlike colonic diverticulosis, ileal and jejunal diverticula are asymptomatic in most cases. No specific clinical signs indicating the presence of jejunal or ileal diverticula have been reported. The clinical presentation is vague and diverse: nonspecific chronic manifestations, such as constipation, dyspepsia, intermittent abdominal pain, diarrhea, and malnutrition, and sometimes acute symptoms such as intra-abdominal abscess perforation (covered or uncovered), diverticulitis (with or without associated hemorrhage), and gastrointestinal obstruction. Terminal ileal diverticulitis mimics appendicitis as a rare cause of pain in the right iliac fossa of the abdomen.

The diagnosis of ileal diverticulitis is often difficult and relies mainly on imaging. A late diagnosis can be deadly, since perforation carries a high risk of mortality, affecting up to 40% of patients [12]. Jejunum-ileum diverticula are discovered incidentally in 75% of cases during laparotomy, autopsy, or barium X-ray [13]. The main differential diagnoses include perforation by a foreign body, neoplasms (with or without perforation), Crohn's disease, traumatic hematoma, and drug-induced ulceration (nonsteroidal anti-inflammatory drugs).

The ileum is difficult to examine using endoscopy; however, the diagnostic tool of choice for these diverticula is imaging [14]. Ileal diverticulosis can be detected by abdominal ultrasound. However, this diagnostic tool has certain limitations such as the presence of adiposity or bloating. Abdominal CT scan is the diagnostic tool of choice, but it is not possible to identify all diverticula in the small intestine. Treatment for ileal diverticulitis is generally conservative [9]. Antibiotics and analgesics are usually sufficient. Surgery is performed in the event of complications [9].

## Conclusions

Although ileal diverticulitis is rare, it should be included in the differential diagnosis of all cases of right lower quadrant pain. Knowledge of the ultrasound and CT scan characteristics of acute terminal ileal diverticulitis enables radiologists to diagnose it accurately, allowing patients to be treated successfully with conservative management and reducing the mortality rate in the event of complications.
